# Colorectal cancer care in Tanzania: an evaluation of clinical characteristics, treatment patterns and quality metrics at a national cancer referral hospital

**DOI:** 10.3332/ecancer.2025.1991

**Published:** 2025-09-16

**Authors:** Beatrice P Mushi, Summaiya Haddadi, Alita Mrema, Jerry Ndumbalo, Nanzoke Mvungi, Msiba Selekwa, Julius Mwaiselage, Larry Akoko, Yona Ringo, Rohan Luhar, Rebecca DeBoer, Katherine Van Loon, Elia Mmbaga, Geoffrey C Buckle

**Affiliations:** 1 Muhimbili University of Health and Allied Sciences, Dar es Salaam 11101, Tanzania; 2 Ocean Road Cancer Institute, Dar es Salaam 11101, Tanzania; 3 Muhimbili National Hospital, Dar es Salaam 11101, Tanzania; 4 Rush University Medical College, Chicago, IL 60612, USA; 5 University of California, San Francisco, San Francisco, CA 94143, USA

**Keywords:** colonic neoplasms, rectal neoplasms, quality indicators, resource-limited settings

## Abstract

**Background:**

Colorectal cancer (CRC) is a leading cause of cancer morbidity and mortality in Tanzania. Non-metastatic CRC is a potentially curable disease that requires multidisciplinary management. This study aimed to evaluate clinicopathologic characteristics for CRC, treatment patterns and select quality metrics at Ocean Road Cancer Institute from 2014 to 2019, the time period immediately preceding the release of Tanzania’s first National Cancer Treatment Guidelines in 2020.

**Methods:**

Quality metrics were selected a priori based upon existing international quality measures, the newly released Tanzania National Cancer Treatment Guidelines and key stakeholder input. Demographic, clinicopathologic and treatment data were abstracted from medical charts for all adult patients with newly diagnosed non-metastatic CRC presenting to Ocean Road Cancer Institute from 2014 to 2019. A clinician reviewed all case report forms for quality assurance. Patient characteristics, treatment patterns and quality metrics were examined using descriptive analyses.

**Results:**

Of 678 patients with CRC, 421 (62%) had non-metastatic disease. Of those with non-metastatic disease, 92 (22%) had colon cancer, 175 (42%) had rectal cancer and 154 (36%) were classified as CRC primary site not otherwise specified. Most patients with colon cancer (n = 86, 93%) underwent surgical resection. Quality of adjuvant chemotherapy was high for colon cancer, with most patients receiving timely treatment (73% within 8 weeks of surgery) and most (81%) with stage III disease receiving appropriate treatment. Documentation in surgical pathology reports was poor, with only 5 of 78 (6%) documenting examination of >12 lymph nodes. Among rectal cancer patients, use of preoperative chemoradiation (7%) and perioperative chemotherapy (27%) was low for locally advanced disease. Overall, only 42 (24%) of patients with rectal cancer underwent surgery and 42 (24%) received no treatment.

**Conclusion:**

The majority of patients with non-metastatic colon cancer received high-quality care, whereas care delivery was less consistent among patients with rectal cancer. This suggests possible challenges in delivering complex, multidisciplinary care in low-resource settings. These findings will serve as a contemporary benchmark for future evaluations of the Tanzanian National Cancer Treatment Guidelines and their impact on CRC care and outcomes.

## Introduction

Colorectal cancer (CRC) is the fourth most commonly diagnosed malignancy and the fifth leading cause of cancer-related death in sub-Saharan Africa [1]. Robust data on the epidemiology of CRC in sub-Saharan Africa are lacking due to a scarcity of population-based cancer registries; however, emerging evidence suggests that CRC rates are rising rapidly across sub-Saharan African countries [2, 3]. This trend mirrors those seen more broadly across low- and middle-income countries (LMICs) worldwide [4]. The rising incidence in LMICs is thought to be driven by epidemiologic and demographic transitions and to changes in modifiable risk factors associated with increasing urbanisation, such as the adoption of Western-pattern diets, increasingly sedentary lifestyles and increases in obesity [5]. Over the next two decades, the burden of CRC in sub-Saharan Africa is projected to rise dramatically to over 100,000 new cases and nearly 75,000 deaths each year [1].

Recent studies have demonstrated significant disparities in CRC survival rates in sub-Saharan Africa compared to high-income settings. In sub-Saharan Africa, estimates of 5-year overall survival among incident cases range from 28% to 48% [6, 7]. By contrast, 5-year overall survival in the United States is 65% [8]. While this ‘outcome gap’ can be partially explained by the higher proportion of patients diagnosed with later stage disease in sub-Saharan Africa – due to delays in diagnosis and the lack of population-based screening programs – treatment access and quality are likely contributing factors as well [9, 10]. A recent analysis of CRC patients identified from population-based care registries demonstrated sub-optimal survival across all stages, including patients with earlier stage, potentially curable disease [7]. For example, 5-year overall survival for stage I/II CRC was 51%–74% [7]. Within the United States, 5-year overall survival for this subgroup is 91% [8]. While these data suggest treatment may play an important role in addressing disparities in CRC outcomes in sub-Saharan Africa, there has been limited research into CRC care in the region. To the best of our knowledge, only one study has examined the quality of CRC treatment and its influence on outcomes. In a study of CRC patients identified from population-based cancer registries in 11 countries, treatment varied widely between hospitals and many patients received care that deviated from leading cancer treatment guidelines [11]. These findings highlight a critical need to develop and evaluate strategies to optimise CRC care in sub-Saharan Africa.

The objective of the present study is to evaluate clinical practice patterns and quality metrics for patients with non-metastatic CRC at Ocean Road Cancer Institute (ORCI), a national cancer referral hospital in Tanzania. The study period focuses on a 5-year time period immediately prior to the release of Tanzania’s first National Cancer Treatment Guidelines (TNCTG) in 2020 [12]. The TNCTG, released by the Tanzania Ministry of Health (MoH), aims to outline a standardised approach for diagnostic work-up and clinical management of CRC and other leading cancers in Tanzania. This study is intended to serve as a benchmark for future investigations into the impact of the TNCTG on CRC care.

## Methods

### Study design

This was a retrospective chart review of all adult patients with newly diagnosed, histologically confirmed, non-metastatic colon and rectal cancer who presented to ORCI from 2014 to 2019. The study time period reflects the 5 years immediately prior to the release of the TNCTG and the launch of a coordinated strategy to implement the guidelines at ORCI beginning in 2020 [12, 13].

### Study setting

The retrospective chart review was conducted at ORCI, a public, tertiary, specialised cancer care facility in Dar es Salaam, Tanzania. ORCI serves as a national cancer referral hospital offering chemotherapy and radiotherapy as treatment options for patients with CRC. ORCI is closely affiliated with Muhimbili National Hospital (MNH), a public teaching hospital and national referral hospital. While ORCI does not offer surgical or endoscopic services on site, patients needing colonoscopy and/or surgery are typically referred to MNH for this care.

### Study population

Patients seen at ORCI from 2014 to 2019 who were aged >18 years and presenting with a new diagnosis of histologically confirmed colonic adenocarcinoma, rectal adenocarcinoma or colorectal adenocarcinoma (with no primary site specified) were considered eligible. Potentially eligible cases were identified through a careful review of ORCI medical records and a search of the ORCI electronic cancer registry using the International Classification of Disease codes. Exclusion criteria included non-permanent residence status, concurrent non-CRC malignancy, two or more synchronous CRC primaries and/or CRC recurrence. No exclusions were made initially based upon staging during the data abstraction phase of the study; however, patients with metastatic disease were subsequently excluded during the quality assurance phase of the study to restrict the analysis to patients with non-metastatic, potentially curable disease.

### Quality metrics

Select quality metrics pertaining to CRC care were chosen a priori based upon existing international quality measures from leading organisations (National Comprehensive Cancer Network, American Society of Clinical Oncology and American College of Surgeons) [14–16], the newly released TNCTG and multidisciplinary input from clinicians and key stakeholders at ORCI and MNH. Key guiding principles that informed the selection of quality metrics included scientific validity (i.e., evidence supporting the quality metric as it relates to CRC outcomes), measurement feasibility/availability in medical charts at ORCI and usability [16]. Quality metrics for colon cancer that were selected were: (i) proportion of patients who initiate adjuvant therapy within 8 weeks of surgery (among patients who undergo surgical resection for localised colon cancer and start adjuvant therapy); (ii) proportion of patients with stage III (node positive) colon cancer who receive adjuvant chemotherapy and (iii) proportion of patients who undergo surgical resection for localised colon cancer and have at least 12 regional lymph nodes removed and pathologically examined. Quality metrics for rectal cancer were: (i) proportion of patients with locally advanced disease who undergo neoadjuvant chemoradiation prior to surgery and (ii) proportion of patients with locally advanced disease who receive perioperative chemotherapy.

### Data collection procedures

A standardised case report form (CRF) was developed and pilot-tested to collect data on baseline clinical information (presenting symptoms, co-morbidities and physical examination findings), investigations (colonoscopy, barium enema, examination under anesthesia, imaging and pathology results), treatment modalities received (surgery, chemotherapy, radiation or a combination of the three treatments) as well as treatment outcomes, if available. The CRFs were designed to collect key clinical information to permit analysis of selected quality metrics.

Trained research assistants (registrars) abstracted the data from the medical chart using paper CRFs, after which data were entered in REDCapÔ, a secure, web-based software platform [17, 18]. All data collected from the medical chart were cross-verified in the ORCI electronic medical record system and treatment logs in the chemotherapy infusion center and the radiotherapy suite.

Following data collection, CRFs were reviewed for quality assurance and to verify subject eligibility. Specifically, CRFs were reviewed to assess the location of the primary tumour (colon, rectal or colorectal not otherwise specified (NOS)) based on the best available data from clinician report, endoscopy, operative reports and/or imaging. Tumours were classified as colorectal NOS if: (i) conflicting information was noted in the medical chart or (ii) tumour was described colorectal or recto-sigmoid without further details. Cancer staging was manually reviewed, with patients classified as non-metastatic disease (i.e., early stage or locally advanced disease) or metastatic disease. Patients with confirmed metastatic disease were excluded from further analyses, with the study focusing on patients who were potentially curable. Cases with missing or conflicting information were reviewed by a second member of the investigator team and adjudicated.

### Statistical analysis

Descriptive analyses were used to examine the sociodemographic and clinicopathologic characteristics and treatments used. Continuous variables were described using median and interquartile ranges and categorical variables using frequency (percentage). Treatment patterns were analysed separately by primary tumour site (i.e., colon versus rectal versus colorectal NOS). For analyses of quality metrics, we reported the proportion of patients who received care that met pre-defined criteria as outlined in the selected quality metrics. Given that quality metrics were defined by primary tumour location, patients were excluded if their primary tumour site could not able to be confirmed.

Statistical analyses were performed using Stata 18 software (StataCorp, College Station, TX) [19]. Sankey diagrams, which are a type of flow diagram that can help visualise treatment pathways (i.e., treatment modalities received and sequence), were created using SankeyMATIC online software [20]. We modeled treatment pathways leading up to and after being seen at ORCI.

### Ethics statement

The institutional review boards at MUHAS (Dar es Salaam, Tanzania), the National Institute for Medical Research of Tanzania and UCSF (San Francisco, California, United States) approved the study.

## Results

A total of 678 patients with newly diagnosed, histologically-confirmed CRC were screened for eligibility. Of these, 421 (62%) had non-metastatic disease at the time of presentation. The sociodemographic characteristics of all eligible patients are summarised in Table 1. Slightly more than half were male (58%). The median age was 51 years (interquartile range 40–63) and 27% were diagnosed at age 45 years or less. The majority (58%) did not have health insurance. Half of the patients reported the Coastal zone as their primary residence.

Clinicopathologic characteristics are summarised in Table 2. Symptoms leading up to diagnosis varied; most common complaints included abdominal pain (47%), hematochezia (36%) and constipation (30%). Only 36% of patients had a colonoscopy at any time point in their care. Most underwent chest X-ray (79%) and abdominal ultrasound (67%) for staging at baseline. Use of magnetic resonance imaging (MRI) (4%) and endoscopic ultrasound (EUS) (0%) was rare. A third of patients did not complete minimum imaging for clinical staging (either chest X-ray and ultrasound or CT scan of chest, abdomen and pelvis).

The primary tumour location was the colon in 92 (22%) and rectal in 175 (42%) patients. In the remaining 154 (37%), primary tumour location was incompletely characterised in the medical charts, herein CRC NOS. Details about the clinicopathologic characteristics and clinical management in patients with non-metastatic CRC NOS are presented in the Appendix.

**Figure 1.**
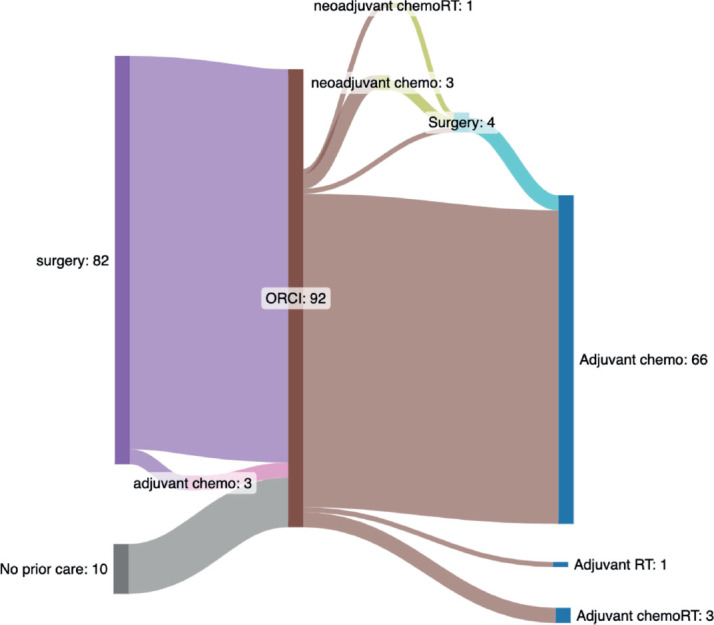
Sankey diagram illustrating observed treatment pathways for patients with non-metastatic colon cancer presenting to Ocean Road Cancer Institute (ORCI). The central vertical bar (labeled ‘ORCI’ and shown in brown) marks the point of presentation toORCI, delineating between care received before ORCI (left-sided nodes) from care received at or after ORCI (right-sided nodes). Each node represents a specific treatment modality and flow lines indicate the number of patients transitioning between treatments. Patients may enter the care pathway at any node (e.g., referred after prior surgery) and stop at any point (e.g., no further treatment). To summarise the left side of the diagram, 82 patients underwent surgery prior to ORCI, including 3 who also received adjuvant chemotherapy. Ten patients presented to ORCI without any prior oncologic treatment. To summarise the right side of the diagram, 70 patients received adjuvant therapy after presenting to ORCI (n = 66 adjuvant chemotherapy, n = 3 chemoradiotherapy, n = 1 radiotherapy). Among the 66 who received adjuvant chemotherapy, 63 had surgery prior to ORCI and 3 underwent surgery after being seen at ORCI. Of the 10 patients without prior treatment at the time they were seen at ORCI (labeled on left side of the diagram as ‘no prior care’), 4 received neoadjuvant therapy at ORCI (n = 3 chemotherapy, n = 1 chemoradiotherapy); 3 of these proceeded to surgery. One of the 10 patients underwent surgery as initial treatment and 5 received no oncologic therapy. Abbreviations: Chemo, chemotherapy; chemoRT, chemoradiotherapy; ORCI, Ocean Road Cancer Institute; RT, radiotherapy.

Table 3 summarises the clinical management of the 92 patients with colon cancer. Most patients (*n* = 86, 93%) underwent upfront surgical resection, with clinical obstruction as the indication in 54 (59%). Surgical pathology reports were available for 78 of 86 (91%) of surgeries. Of these, 8 (10%) noted a positive margin and 5 (6%) reported evaluation of 12 or more lymph nodes. A total of 52 (67%) of pathology reports were missing details on depth of invasion and/or nodal involvement. Nearly three-quarters (74%) of all patients with colon cancer received chemotherapy, most (*n* = 66, 72%) in the adjuvant setting. Radiation was rarely used among patients with colon cancer, with only one patient receiving neoadjuvant chemoradiation and four patients receiving adjuvant radiation with or without chemotherapy. Figure 1 summarises treatment patterns among patients with non-metastatic colon cancer leading up to and following presentation at ORCI, the cancer referral hospital.

Clinical management of the 175 patients with rectal cancer is summarised in Table 4. Sixty-seven (38%) initiated neoadjuvant therapy. Among patients treated with neoadjuvant therapy, chemoradiation alone and chemotherapy alone were the most common treatment approaches used in 34 (51%) and 20 (30%) of these patients, respectively. Only 4 (6%) received sequential chemotherapy alone and chemoradiation in the neoadjuvant setting. A total of 42 (24%) of patients underwent surgery. Surgical pathology reports were available for 88% of these surgeries. Of these, 5 (12%) were noted to have a positive margin. None of the pathology reports reported evaluation of 12 or more lymph nodes. A total of 68 (39%) received some form of adjuvant therapy, with chemoradiation alone (18%) and chemotherapy alone (14%) being most common.

**Figure 2.**
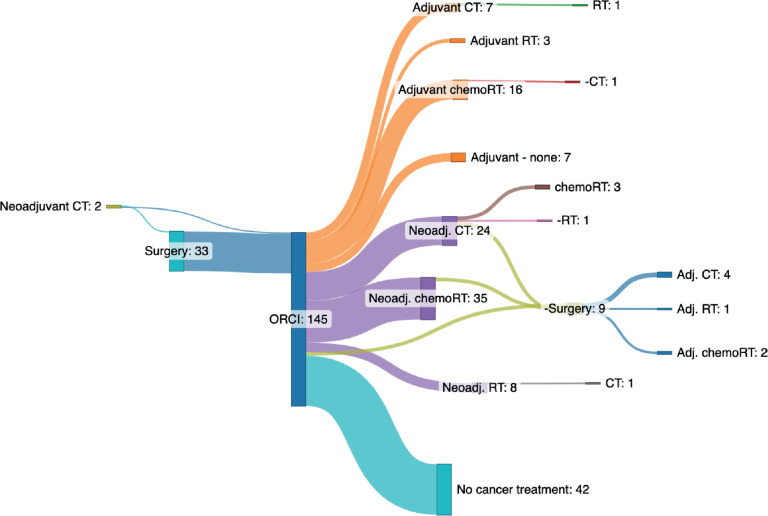
Sankey diagram illustrating treatment pathways for patients with non-metastatic rectal cancer presenting to Ocean Road Cancer Institute (ORCI). The central vertical bar (labeled ‘ORCI’ and shown in royal blue) marks the point of presentation to ORCI, delineating care received before ORCI (left-sided nodes) from care received at or after ORCI (right-sided nodes). Each node represents a distinct treatment modality, and flow lines indicate the number of patients moving between treatments. Patients may enter or exit the care pathway at any node. To summarise the left side of the diagram: a total of two patients received neoadjuvant chemotherapy and 33 underwent surgery (n = 1 neoadjuvant chemotherapy followed by surgery, n = 1 neoadjuvant chemotherapy alone; and n = 32 surgery alone). On the right side: 26 patients initiated adjuvant therapy upon presentation to ORCI (n = 16 chemoradiotherapy, n = 7 chemotherapy and n = 3 radiation therapy). This figure represents 26 of the 33 patients who had surgery prior to ORCI, with 7 of those patients receiving no further therapy. A total of 67 patients initiated neoadjuvant therapy at ORCI as their first treatment (n = 35 chemoradiotherapy, n = 24 chemotherapy, n = 8 radiotherapy); of these, 6 proceeded to surgery (n = 3 after chemoradiotherapy, n = 3 after chemotherapy). An additional 3 patients underwent surgery as their first and only treatment after presenting to ORCI. Overall, 42 patients received no cancer-directed therapy. Abbreviations: CT, chemotherapy; chemoRT, chemoradiotherapy; ORCI, Ocean Road Cancer Institute; RT, radiotherapy. Note: Thirty patients were excluded from this figure due to inconsistent or incomplete documentation regarding surgical care.

Treatment pathways among patients with rectal cancer were heterogeneous. Table 5 summarises the combinations of treatment modalities used among all 175 patients. Only 2 (1%) patients received trimodality therapy (i.e., chemotherapy, chemoradiation and surgery). A total of 24% of patients received no cancer-directed treatment. We mapped out treatment patterns and sequencing for the subset of 145 patients (83%) with well-defined details on cancer-directed therapy (chemotherapy, radiation with or without chemotherapy), surgery and surgical pathology in Figure 2. A total of 30 (17%) were excluded from this analysis due to inconsistencies noted in documentation (e.g., surgery was referenced in the medical chart, but there was limited detail on the type of surgery performed, pathology reports missing entirely or inconsistent reference to treatment ‘setting’ (i.e., neoadjuvant and adjuvant)).

Analysis of select quality metrics for the management of patients with non-metastatic colon and rectal cancer is presented in Table 6. A high proportion of patients with colon cancer met quality metrics pertaining to clinical care, with 17 of 21 (81%) of patients with stage III disease receiving adjuvant chemotherapy and 41 of 56 (73%) of patients who received adjuvant therapy initiating within 8 weeks of surgery. Few patients with available surgical pathology reports (*n* = 5 of 78, 6%) had documentation of 12 or more lymph nodes being removed and pathologically examined. Among patients with rectal cancer, lower proportions of patients met criteria for quality metrics; use of preoperative chemoradiation for locally advanced disease was low (*n* = 3, 7%) and fewer patients received perioperative chemotherapy for locally advanced disease (*n* = 48, 27%).

## Discussion

In the present study, we report clinicopathologic characteristics, treatment patterns and select quality metrics among patients with non-metastatic CRC presenting to ORCI, a national cancer referral hospital in Tanzania. To the best of our knowledge, this study represents one of the largest cohort studies focused on CRC management in East Africa to date, and the first in sub-Saharan Africa to examine quality metrics related to CRC care.

In the present study, we found a high proportion of patients present as clinical emergencies (e.g., obstruction and perforation). This aligns with previous research on CRC in Tanzania [21, 22] and other East African countries [23–25]. Importantly, many patients also reported longstanding antecedent symptoms. These findings suggest there may be opportunities for earlier detection of CRC in Tanzania by promoting greater awareness of clinical signs and symptoms of the disease. In our study, a high proportion (38%) of patients presented with metastatic disease at the time of diagnosis. This figure aligns with previous case series in Tanzania (25%–38%) [22, 21] and data from the African Cancer Registry Network (31%) [11], but exceeds figures reported in the US and other high-income settings (typically ranging ~20% [26–28]). This difference is not surprising given the lack of population-based CRC screening in Tanzania and sub-Saharan Africa more broadly.

In our study, analysis of treatment patterns identified several key findings. Most patients diagnosed with localised colon cancer underwent upfront surgery followed by chemotherapy. This management approach aligns with leading international guidelines and the TNCTG, which were released after the study period. Care delivery for patients with rectal cancer, by contrast, was highly varied. Among patients diagnosed with locally advanced disease, a total of 11 different combinations of treatment modalities were used. Notably, few patients received trimodality therapy (i.e., the combination of chemotherapy, chemoradiation and surgery), a treatment approach that was widely accepted as standard of care during the study period. Surgery in this patient population was uncommon, with only 24% undergoing surgery at any point during their treatment course. An equal proportion received no therapeutic intervention at all. This low proportion of patients with rectal cancer undergoing trimodality care – and surgery in particular – identifies that optimising multi-disciplinary care for patients with locally advanced rectal cancer in Tanzania is an unmet need.

Analysis of quality metrics demonstrated mixed results in our study. Quality metrics assessing care delivery among colon cancer patients indicated high-quality care. Most patients received timely adjuvant therapy (73%) and adjuvant therapy use was high (81%) in a subgroup at high risk for recurrence (node-positive or stage III disease). While these findings reflect favourably, we found suboptimal reporting/documentation of pathology results for the surgical resection of localised colon cancer. Only 5% of surgical pathology reports documented 12 or more lymph nodes examined. This finding raises concern that a subset of patients who undergo surgical resection for localised colon cancer may be under-staged and those documented as having stage II disease may have undetected node-positive/stage III disease. This could have important implications for treatment in impacted patients, particularly given robust evidence indicates adjuvant therapy has a greater magnitude of benefit in stage III versus stage II disease [29, 30].

In this study, quality metrics for rectal cancer demonstrated less consistency in care as compared to colon cancer. Specifically, we found low rates of preoperative chemoradiation (7%) and perioperative chemotherapy (27%) for patients with locally advanced disease. Based upon prior studies in locally advanced rectal cancer, our findings suggest patients may face increased risk of local recurrence (due to low rates of preoperative chemoradiation [31, 32]) and systemic recurrence (due to low rates of perioperative chemotherapy [33]). However, contextualising these findings within other care patterns seen in patients with locally advanced disease – specifically, low rates of surgery, high rates of patients receiving no cancer-directed treatment and few receiving trimodality therapy – suggests there are likely multiple barriers throughout the care pathway for this patient population.

Several factors could explain the differences in quality metrics between colon and rectal cancer in our study. First, important differences exist in clinical management. This is largely due to the technical challenges of surgical resection in rectal cancer and the increased risk of local recurrence. This can be explained by tumour proximity to pelvic structures, the lack of serosa covering the rectal tissue and the challenges of achieving negative margins. Preoperative chemoradiation has emerged as a standard treatment to address this risk, as it has been shown to decrease the likelihood of local recurrence. This additional treatment modality creates additional complexity in care for patients with rectal cancer. It also may introduce additional logistical, financial and/or cultural barriers to care. Some of the variation in the rectal cancer management in our study may be explained by these barriers, others specific to radiation or the need for treatment prior to surgery. Second, differences in clinical staging at the time of presentation may also help explain our findings. Clinicians at ORCI report that it is not uncommon for patients with rectal cancer to present with unresectable primary tumours. This determination informs treatment decisions, with a subset of patients recommended for induction chemotherapy with the goal of ‘conversion’ to resectability, though in some cases, patients may not experience tumour shrinkage to justify continued treatment with curative intent. In other cases, patients are recommended for care that is largely directed toward palliation, given that the primary tumour is viewed as ‘unlikely to become resectable.’ Clinician assessment of ‘resectability’ of primary tumours was not routinely available in medical charts at ORCI, and therefore, this detail could not be accounted for in analyses. These findings – and the limitations in interpreting these results – highlight a need for prospective studies to examine clinical care delivery and barriers to care for patients with CRC.

Few previous studies have examined CRC care delivery at other African centers. In one study, researchers leveraged the African Cancer Registry Network to examine CRC treatment patterns and alignment with the new National Comprehensive Cancer Network (NCCN) Harmonised Guidelines for Sub-Saharan Africa for Colon and for Rectal Cancer [34, 35] in a subset of patients identified from population-based cancer registries [11, 36]. While this study is notable for being among the first to evaluate ‘guideline concordant care’ for CRC in sub-Saharan Africa, direct comparisons with our study are limited due to two differences in their methodologic approach: (i) the study aggregated the outcomes of colon and rectal cancer and (ii) deviations in care were not reported in detail, but rather grouped in categories of deviation (major, minor and none). In this study, only 3% of patients with non-metastatic CRC received guideline-concordant care, 21% received guideline-concordant care with minor deviations (e.g., neoadjuvant chemoradiation for rectal cancer using non-fluoropyrimidine-based chemotherapy), 32% with major deviations (e.g., for stage III colon cancer, incomplete surgical resection and/or incomplete chemotherapy) and 35% received no form of treatment [11]. Similar to our study, reasons for the low receipt of guideline-concordant care were outside the scope of analysis, but possibilities cited included patient attitudes (lack of awareness of modern medicine, preference for alternative therapy, fear of side effects), stigma (related to cancer and/or colostomy bags) as well as health-system factors (e.g., limited availability of surgeons). Importantly, this study found a strong correlation between receipt of guideline-concordant care and overall survival, suggesting improvements in the delivery of care quality have the potential to improve patient outcomes. These results, along with our findings, call for further investments in implementation research to fully investigate multi-level barriers to CRC care in sub-Saharan Africa.

Our study has several important limitations. Our analysis yielded a high number of patients (37%) for whom the exact location of the primary tumour could not be determined due to incomplete information in the medical chart. Some of these patients likely had rectosigmoid primaries. However, there was insufficient information to discern those patients from others who were described as having colorectal tumours, with location not specified. This figure is comparable to previous CRC research in Tanzania conducted with retrospective chart review [22], yet highlights the limitations of relying on medical charts for health service research. Some challenges with classifying patients could also be attributed to the low proportion of patients undergoing critical diagnostic studies. Few underwent colonoscopy and proctoscopy, a finding that has been described previously, and this introduced challenges in classifying patients as having colon versus rectal tumours [22, 21]. In addition, a few patients with rectal tumours underwent rectal MRI and EUS. MRI or EUS is generally viewed as necessary to differentiate patients from early stages from locally advanced tumours. Additionally, half of the patients in our study reported the Coastal zone as their primary residence. While this finding may suggest geographic variability in incidence rates – or a higher burden of CRC in the Coastal zone compared to others – we suspect this most likely reflects referral patterns and access to care, as ORCI is located within the Coastal zone and serves as the primary cancer referral center for this geographic area. Importantly, clinicopathologic characteristics and care patterns for CRC may differ at centers in other regions across the country. Nearly a third of patients did not undergo complete staging with chest radiograph and ultrasound or cross-sectional imaging. Importantly, this lack of information introduces limitations to our study and also poses major challenges for clinical decision-making. The analysis of care delivery was limited by the quality and incompleteness of the pathology reports, specifically the lack of detail regarding depth of invasion, nodal involvement and clinicopathologic features that are prognostic factors. These findings call for dedicated efforts focused on introducing synoptic reporting in surgical pathology for CRC to improve care delivery. Finally, long-term outcomes of treatment were not routinely available due to the retrospective study design.

This study offers important insights into clinical care for patients with CRC in Tanzania and other similar low-resource settings, with several key findings. We demonstrated that many patients with colon cancer received high-quality care, with successful surgery and most receiving timely adjuvant chemotherapy. By contrast, rates of preoperative chemoradiation and perioperative chemotherapy for locally advanced rectal cancer were low and few received standard trimodality therapy. There is a critical need for further research to investigate barriers to care for this patient population. In addition, our study highlighted major gaps in the completeness of diagnostic studies and clinical documentation. These gaps have the potential to adversely impact care delivery when full data is not available to inform clinical decision-making. Interventions are needed to standardise documentation, particularly in a context in which multi-disciplinary cancer care must be coordinated across different institutions.

Looking ahead, a prospective observational cohort study is underway at ORCI evaluating clinical care, patient health outcomes and quality metrics among patients diagnosed with CRC from 2020 to 2022. This time period follows the release of the TNCTG. This study will serve as a historic benchmark for future evaluation of the impact of the TNCTG – and a coordinated guideline implementation strategy – on standardising CRC care and improving patient outcomes. More so, our approach of embedding quality metrics has the potential to serve as a model for future efforts aimed at promoting evidence-based CRC care.

## List of abbreviations

BMC, Bugando Medical Center; CRC, Colorectal cancer; CRF, Case report form; EUS, Endoscopic ultrasound; HICs, High-income countries; HIV, Human immunodeficiency virus; ICD, International Classification of Disease; IQR, Interquartile Range; KCMC, Kilimanjaro Christian Medical Centre; LMICs, Low- and middle-income countries; MNH, Muhimbili National Hospital; MoH, Ministry of Health; MRI, Magnetic resonance imaging; MUHAS, Muhimbili University of Health and Allied Sciences; NCCN, National Comprehensive Cancer Network; NOS, Not otherwise specified; ORCI, Ocean Road Cancer Institute; TNCTG, Tanzania National Cancer Treatment Guidelines; UCSF, University of California, San Francisco.

## Conflicts of interest

The authors declare that they have no conflict of interest.

## Previous publication

This study was presented as a poster at the African Organization for Research and Training in Cancer (AORTIC) 2023 Meeting in Dakar, Senegal.

## Figures and Tables

**Table 1.** Sociodemographic characteristics of patients with newly diagnosed, non-metastatic colorectal cancer attending Ocean Road Cancer Institute, Dar es Salaam, Tanzania from 2014 to 2019.

Characteristics	*N* (%)
Total	421
Gender	
Male	243 (58)
Female	177 (42)
Age, median (IQR)*	51 years (40–63)
< 45 years	114 (27)
> 45 years	205 (49)
Zones	
Northern zone	69 (16)
Coastal zone	209 (50)
Central zone	30 (7)
Southern Highlands	29 (7)
Southwest Highlands	28 (7)
Other	50 (12)
Health Insurance status	
Insured	179 (42)
Not insured	242 (58)
Comorbidities	
Diabetes	27 (6)
Hypertension	48 (11)
HIV	14 (3)

* Age missing for 24% (102) study participants

*IQR* interquartile range

**Table 2.** Clinical characteristics and diagnostic investigations among patients with non-metastatic colorectal cancer.

	*N* (%)
Total	421
Duration of symptoms at time of presentation, median (IQR)	6 months (3–12 months)
Presenting symptoms	
Change in bowel habits	118 (28)
Constipation	127 (30)
Blood in stools	155 (36)
Abdominal pain	196 (47)
Weakness/Fatigue	61 (14)
Weight loss	50 (12)
Colonoscopy performed	149 (36)
EUS	0 (0)
Obstruction noted during work up*	62 (15)
Imaging	
Chest X-ray	332 (79)
Abdominal ultrasound	282 (67)
Computed tomography scan	182 (43)
MRI	15 (4)
Histological confirmation	421 (100)
Diagnosis	
Colon cancer	92 (22)
Rectal cancer	175 (42)
Colorectal cancer NOS	154 (37)

* Based upon any one or more of the following investigations: colonoscopy, barium enema or exam under anesthesia; IQR interquartile range, NOS not otherwise specified

**Table 3.** Clinical management of patients with non-metastatic colon cancer.*

	*N* (%)
Total	92 (100)
Surgery	
Surgery performed	86 (93)
Colectomy	74 (80)
Transabdominal radical resection	7 (8)
Primary resection (NOS)	5 (5)
Surgical indication	
Obstruction	54 (59)
Perforation	2 (2)
Not documented	36 (39)
Surgical pathology	
Available	78 (91)
Margins	
Positive	8 (10)
Not documented	21 (27)
>/= 12 LN evaluated	5 (6)
Pathological staging	
TX NX	10
TX N0	3
TX N1	2
T1/T2 NX	27
T1/T2 N0	4
T1/T2 N1	8
T1/T2 N2	4
T3 NX	6
T3 N0	3
T3 N1	5
T4 NX	4
T4 N1	2
Chemotherapy	*N* (%)
Perioperative chemotherapy (any)	68 (74)
Neoadjuvant	3 (3)
Adjuvant	66 (72)
Mean number of cycles	8.90 (SD 3.87)

CT computed tomopgraphy LN lymph nodes NOS not otherwise specified SD standard deviation; * A total of 63 (68%) completed minimum imaging for staging (defined as imaging of the chest, abdomen and pelvis using any combination of CT, chest X-ray and/or abdominal/pelvic ultrasound)

**Table 4.** Clinical management of patients with non-metastatic rectal cancer^a,b^.

	*N* (%)
Total	175 (100)
Neoadjuvant therapy	67 (38)
Chemotherapy alone	20 (30)
Chemoradiation alone	34 (51)
Radiotherapy alone	7 (10)
Chemotherapy -> Chemoradiation	4 (6)
Radiotherapy -> Chemotherapy	1 (2)
Chemotherapy -> Radiotherapy	1 (2)
Surgery	42 (24)
Transabdominal resection	29 (17)
Primary resection (NOS)	9 (5)
Trans-anal excision	4 (2)
Surgical pathology	
Available	37 (88)
Margins	
Positive	5 (12)
Not documented	18 (43)
>/= 12 LN evaluated	0 (0)
Pathological staging	
TX NX	15 (36)
TX N1	1 (2)
TisN2	1 (2)
T1/T2 NX	13 (31)
T1/T2 N0	2 (5)
T1/T2 N1	1 (2)
T3 NX	1 (2)
T3 N0	2 (3)
T3 N1	1 (2)
T3 N2	1 (2)
T4 NX	1 (2)
T4 N2	2 (5)
Adjuvant therapy	
Chemotherapy alone	25 (14)
Radiotherapy alone	9 (5)
Chemoradiation alone	32 (18)
Chemotherapy -> Radiotherapy	1 (1)
Chemoradiation -> Chemotherapy	1 (1)

CT computed tomopgraphy NOS not otherwise specified

a A total of 122 (69%) completed minimum imaging for staging (defined as imaging of the chest, abdomen and pelvis using any combination of CT, chest X-ray and/or abdominal/pelvic ultrasound)

b A total of 6 (3%) underwent pelvic MRI or EUS for clinical staging of primary tumour

**Table 5.** Overall treatment patterns for patients with non-metastatic rectal cancer.

	*N* (%)
Total	175 (100)
Chemotherapy + Chemoradiation + Surgery (trimodality care)	2 (1)
Chemotherapy + Chemoradiation	7 (4)
Chemotherapy + Radiotherapy	2(1)
Chemotherapy + Surgery	10 (5)
Chemoradiation + Surgery	19 (11)
Radiotherapy + Surgery	5 (3)
Chemotherapy alone	27 (15)
Radiotherapy alone	12 (7)
Chemoradiation alone	43 (25)
Surgery alone	6 (3)
No therapy	42 (24)

**Table 6.** Quality metrics for non-metastatic colorectal cancer.

Metric	*N* (%)
Colon cancer	
>12 lymph nodes removed and pathologically examined for resected colon cancer^a^	5 (6)
Adjuvant therapy for patients with stage III colon cancer^b^	17 (81)
Initiated adjuvant therapy within 8 weeks of surgery^c^	41 (73)
Rectal cancer^d^	
Neoadjuvant chemoradiation before surgery for locally advanced disease^e^	3 (7)
Perioperative chemotherapy for locally advanced disease	48 (27)

a Among 78 patients with surgical pathology available; surgical pathology was missing for 8 patients

b Defined as patients with stage III disease based upon surgical pathology (total *n* = 21)

c Among 56 patients who received adjuvant therapy with available dates of surgery and of initiation of adjuvant therapy. *N* = 10 were missing dates of surgery and/or adjuvant therapy

d All 175 patients with rectal cancer either met criteria for locally advanced disease (i.e., T3 or N+) or lacked sufficient diagnostic work up with pelvic MRI and/or EUS to diagnose as T1 or T2 N0 to support local intervention (i.e., upfront surgical resection via transabdominal resection or transanal excision)

e Among all 42 patients who underwent surgery

## Appendix

**Table S1.** Clinical management of patients with non-metastatic CRC NOS.

	*N* (%)
Total	154
Neoadjuvant therapy	8 (5)
Chemotherapy followed by chemoradiation	1 (1)
Chemoradiation	3 (2)
Chemotherapy	4 (3)
Surgery	105 (68)
Colectomy	69 (45)
Transabdominal resection	29 (18)
Total proctocolectomy	1 (1)
Transanal excision	6 (4)
Surgical indication	
Obstruction	84 (80)
Perforation	2 (2)
Adjuvant therapy	
Chemotherapy	81 (53)
Chemotherapy and radiation (sequential)	1 (1)
Radiotherapy	3 (2)
Concurrent chemoradiation	14 (9)

**Table S2.** Clinical management of patients with non-metastatic CRC NOS.

	*N* (%)
Total	154
Neoadjuvant therapy	8 (5)
Chemotherapy followed by chemoradiation	1 (1)
Chemoradiation	3 (2)
Chemotherapy	4 (3)
Surgery	105 (68)
Colectomy	69 (45)
Transabdominal resection	29 (18)
Total proctocolectomy	1 (1)
Transanal excision	6 (4)
Surgical indication	
Obstruction	84 (80)
Perforation	2 (2)
Adjuvant therapy	
Chemotherapy	81 (53)
Chemotherapy and radiation (sequential)	1 (1)
Radiotherapy	3 (2)
Concurrent chemoradiation	14 (9)
